# Convention for the Revision of the Pharmacopœia

**Published:** 1850-03

**Authors:** 


*Convention for the Revision of the Pharmacopœia.*—Dr. Charles A. Lee has been elected by the Faculty of the Medical College of Buffalo, delegate to the National Medical Convention, for the revision of the Pharmacop[illegible]a, which meets at Washington on the first Monday in May, 1850.

